# Suppository Bases, Especially for the Tropics

**Published:** 1910-07-16

**Authors:** 


### THERAPEUTICS.

### SUPPOSITORY BASES, ESPECIALLY FOR THE TROPICS.

While cacao butter is without doubt a very satisfactory suppository base, it, generally speaking, is unsuitable when used in southern latitudes. Suppositories prepared with cacao butter as the base will not stand exposure to the summer heat. On very hot days they are apt to be in a bad condition when received by customers, even when sent directly from the iced moulds, depending of course on the distance sent, or, rather, upon the time exposed.

Again, it proves unsatisfactory when combined with a large percentage of solid extracts—5 grains, say, to the 30-grain suppository. Particularly is this true of extract of witch-hazel. The fault here is apparently because of the fact that when liquefied the fluidity of cacao butter is great; it lacks viscosity, and unless kept very near the congealing point, a more or less difficult proposition, the extracts will separate from the butter either in fine particles or sometimes as a mass.

Of course, in searching for a substitute to be used when it is desired to combat the difficulties mentioned, it must be borne in mind that the substance must melt at about 98° F. Several years back Mr. H. A. B. Dunning in America recommended the use of cacao butter containing 15 per cent. of castor oil and 10 per cent. of white wax as a substitute for cacao butter alone for summer use. Having had a complaint about some adrenalin suppositories prepared with this base, he was led to make the following investigation. Although the mode of testing is doubtless very crude, he thinks the comparative results are fairly satisfactory and somewhat instructive.

The chief test was carried out by holding suppositories of cacao butter alone, and cacao butter in various combinations in the hand and noting the effects. In several cases the capacity for heat was observed in the following crude but comparatively accurate manner: A definite weight of paraffin was kept liquid on a sand bath; in the paraffin was suspended a definite quantity of water in a beaker, which was kept at a constant temperature. In testing the suppository by this method it was floated in the heated water and the time required for it to melt was noted.

Cacao butter suppository, melting-point 33° C. or thereabouts, when held in the hand is softened in three minutes and melts in six, running freely and spreading rapidly over a large surface. When tested by the warm-water method it requires three minutes to liquefy.

Cacao butter with 15 per cent. of castor oil, m.p. 32° C., held in the hand is softened in about three minutes, melts in five, runs freely and spreads rapidly, and is less liquid than plain cacao butter. With the warm-water method it melts in two minutes and forty seconds. Cacao butter with 15 per cent. of castor oil and 10 per cent. of white wax held in the hand softens in ten minutes, becomes creamy in twenty minutes, does not run or spread well, but has a tendency to hold its shape. Cacao butter with 15 per cent. castor oil and 5 per cent. of white wax, m.p. 36° C. or thereabouts, held in the hand softens in about ten minutes, becomes creamy in seventeen minutes, and otherwise acts very much as the preceding combination does. Cacao butter with castor oil 10 per cent., white wax 2½ per cent., m.p. 34° C. or thereabouts, held in the hand softens in eleven minutes, melts in sixteen minutes, runs freely and spreads nicely. With warm water it liquefies in four and three-quarter minutes.

For use in summer-time, and when a large percentage of solid extracts is dispensed, a base composed of cacao butter containing castor oil 10 per cent. and white wax 2½ per cent. is more satisfactory than cacao butter alone. This mixture melts at body temperature and spreads quite freely.

The addition of one-twenty-fourth of a grain of adrenalin to a 30-grain suppository increases the resistance of cacao butter to heat and prevents the suppository from melting freely at the body temperature.

